# Increased FURIN expression in rheumatoid arthritis patients and its anti‐inflammatory effect

**DOI:** 10.1002/jcla.23530

**Published:** 2020-08-25

**Authors:** Rong Cao, Ying Zhang, Juping Du, Shuaishuai Chen, Na Wang, Haijian Ying, Bo Shen

**Affiliations:** ^1^ School of Laboratory Medicine and Life Sciences Wenzhou Medical University Wenzhou China; ^2^ Department of Clinical Laboratory Taizhou Enze Medical Center (Group) Taizhou Hospital of Zhejiang Province Taizhou China

**Keywords:** DAS28, FURIN, rheumatoid arthritis, THP‐1 derived macrophages

## Abstract

**Background:**

FURIN belongs to the proprotein convertase family that processes proproteins and is involved in many diseases. However, the role of FURIN in rheumatoid arthritis (RA) remains unknown. In this study, we investigated the association between circulating FURIN and disease activity in patients with RA and the effect of FURIN in THP‐1‐derived macrophages.

**Methods:**

A total of 108 RA patients and 39 healthy controls participants were included in this study. RA patients were divided into four disease activity groups determined by the Disease Activity Score of 28 joints (DAS28). FURIN expression in peripheral blood mononuclear cells (PBMCs) and serum was detected by using quantitative real‐time polymerase chain reaction (qRT‐PCR) and enzyme‐linked immunosorbent assay (ELISA), respectively. Western blotting and qRT‐PCR were used to detect cytokines level after interfering FURIN expressed in THP‐1‐derived macrophages.

**Results:**

Both FURIN mRNA and protein levels were significantly higher in RA patients than in healthy controls participants (*P* < .001). No significant difference in FURIN expression was observed among the four RA groups (*P* > .05). Spearman correlation revealed that FURIN positively correlated with transforming growth factor‐β1(TGF‐β1), rheumatoid factor (RF), and anti‐cyclic citrullinated peptide (anti‐CCP). Moreover, the inhibition of FURIN in THP‐1‐derived macrophages promoted the caspase‐1 and IL‐1β expression (*P* < .05).

**Conclusion:**

FURIN levels were significantly increased in the peripheral blood of RA patients and were not associated with disease activity. The inhibition of FURIN in THP‐1‐derived macrophages with elevated IL‐1β levels shows that FURIN may have an anti‐inflammatory effect.

## Graphical abstract

FURIN inhibition in THP‐1‐derived macrophages upregulated IL‐1β and caspase‐1. To investigate the effects of FURIN on cytokine secretion in THP‐1 derived macrophages, we used siRNA to interfere with the expression of FURIN (si‐FURIN group), followed by incubation with LPS (1 μg/mL) for 4 hours and ATP (5 mmol/L) for 30 minutes that could then be used to detect changes in the levels of several cytokines, including TNF‐α, IL‐1β, and TGF‐β1. (A). Western blot of THP‐1‐derived macrophages transfected with either FURIN siRNA (si‐FURIN) or control siRNA (NC) for 24 h. (B). FURIN protein levels. (C). FURIN mRNA levels. (D). TGF‐β1 mRNA levels. (E). IL‐1β mRNA levels. (F). TNF‐α mRNA levels. (G). Western blot of THP‐1‐derived macrophages transfected with either FURIN siRNA (si‐FURIN) or control siRNA (NC) for 24 hours, followed by incubation with LPS (1 μg/mL) for 4 hours and ATP (5 mmol/L) for 30 minutes. (H). FURIN protein levels; (I). IL‐1β protein levels. *P < .05, **P < .01.

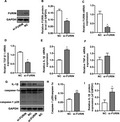

## 1 INTRODUCTION

Rheumatoid arthritis (RA) is an autoimmune disease characterized by joint inflammation. Its clinical manifestations include progressive and destructive polyarthritis associated with serological evidence of autoreactivity. One of the pathological causes of RA is hyperactive immune cell responses that result in elevated levels of inflammatory cytokines. Evidence suggests that inhibitors of special cytokines, for example, TNF‐α, IL‐1, IL‐17, and IL‐6, provide improvement in RA patients.^1^, ^2^

FURIN is one of the nine members of the proprotein convertases subtilisin/kexin (PCSK) family and cleaves various precursor proteins in several physiological processes.^3^, ^4^ FURIN is involved in regulating the secretion of cytokines, is usually co‐expressed with transforming growth factor‐β1 (TGF‐β1), and is a vital regulator of the TGF‐β1 cytokine.^5^, ^6^ Furthermore, T cell‐expressed FURIN is essential for the maintenance of peripheral immune tolerance and is critical for overall health and regulatory function of the body.^7^ Thus, the dysregulation of FURIN levels may lead to different diseases and disorders,^8^ such as primary Sjogren's syndrome disease (pSS), systemic lupus erythematosus (SLE), and RA.^9^, ^10^, ^11^ A study on RA demonstrated, the overexpression of FURIN in fibroblast‐like synoviocytes (FLS).^9^ Further, systemic administration of exogenous FURIN in mice with collagen‐induced arthritis (CIA) mitigates the symptoms of RA.^12^

However, the relationship between circulating FURIN and the disease activity of RA is unclear. Hence, we studied peripheral blood mononuclear cells (PBMCs) and serum FURIN levels in RA patients and healthy controls participants, and analyzed the correlations between FURIN expression and RA disease activity in a Chinese population. Moreover, the effect of FURIN on cytokines (IL‐1β, TNF‐α, and TGF‐β1) production was also studied in THP‐1‐derived macrophages.

## 2 MATERIALS AND METHODS

### 2.1 Study population

Peripheral blood samples of 108 (75 female and 33 male) RA patients and 39 (27 female and 12 male) healthy controls participants were collected from September 2018 to December 2018. The diagnosis of RA at Taizhou Hospital, Zhejiang Province, China, was based on the 2010 American College of Rheumatology (ACR)/European League Against Rheumatism (EULAR) criteria.^13^ Patients with other persistent inflammatory diseases, autoimmune diseases, or cancers were excluded. Clinical data were obtained from electronic medical record. We also requested for information about the medications taken by the respective patients within 2 months prior to this study. The disease activity of RA patients was assessed by using the Disease Activity Score of 28 joints (DAS28). All RA patients were divided into four groups: high disease activity group (DAS28 > 5.1), moderate disease activity group (3.2 < DAS28 ≤ 5.1), low disease activity group (2.6 < DAS28 ≤ 3.2), and remission group (DAS28 ≤ 2.6). The study was approved by the Medical Ethics Committee of Taizhou Hospital of Zhejiang Province, and informed consent was obtained from each patient.

### 2.2 Clinical laboratory data collection

Complete blood count of samples was performed with BC‐6800Plus (Mindray), and erythrocyte sedimentation rate (ESR) was obtained by using a fully automated analyzer (Alifax Test 1). Anti‐cyclic citrullinated peptide (anti‐CCP) was quantified by using immunoturbidimetric assay i2000 (Architect, American). The concentrations of rheumatoid factor (RF), C‐reactive protein (CRP), immunoglobulins IgM, IgA, IgG, and C3 and C4 were measured with an AU5800 (Beckman Coulter, American).

### 2.3 Quantitative reverse transcriptase real‐time polymerase chain reaction (qRT‐PCR)

Total RNA was extracted using TRIzol™ reagent (15596018, Life Technologies) according to standard operating procedures. BeyoRT First Strand cDNA Synthesis kit (D7168M, Beyotime) was used for the reverse transcription total RNA into complementary DNA (cDNA). qRT‐PCR assay of cDNA was performed using FastStart Universal SYBR Green Master (ROX) (Roche). We analyzed the association between FURIN and TGF‐β1 expression in the peripheral blood of RA patients. The mRNA levels of FURIN and TGF‐β1 were normalized according to the expression levels of the glyceraldehyde‐3‐phosphate dehydrogenase housekeeping gene *GAPDH*. The relevant primer sequences used in this study are shown in Table 1.

**Table 1:** The primer sequence

Gene name	Primer	Sequences (5′‐3′)
FURIN	Forward	GCAAAGCGACGGACTAAACG
	Reverse	TGCCATCGTCCAGAATGGAGA
TGF‐β1	Forward	CAATTCCTGGCGATACCTCAG
	Reverse	GCACAACTCCGGTGACATCAA
TNF‐α	Forward	GAGGCCAAGCCCTGGTATG
	Reverse	CGGGCCGATTGATCTCAGC
IL1β	Forward	AGCTACGAATCTCCGACCAC
	Reverse	CGTTATCCCATGTGTCGAAGAA
GAPDH	Forward	TGGACCTGACCTGCCGTCTA
	Reverse	GGAGTGGGTGTCGCTGTTGA

### 2.4 Enzyme‐linked immunosorbent assay (ELISA)

Serum samples were stored at −80°C until the levels of the cytokines were detected. FURIN was detected in 108 RA patients and 39 healthy control participants by using a commercial Human FURIN Enzyme‐Linked Immunosorbent Assay (ELISA) Kit (Sigma Aldrich, USA), and the results are presented as pg/mL. The concentrations of IL‐1β, IL‐4, IL‐6, TNF‐α, and IL‐10 were determined by using ELISA kits (Mlbio) and TGF‐β1 ELISA kits (Elabscience) according to the manufacturer's instructions.

### 2.5 Transient transfection of siRNA in THP‐1‐derived macrophages

THP‐1 monocytes cell line was obtained from China Center for Type Culture Collection (Wuhan, China). Cells were seeded in 12‐well plates at a density of 5 × 10^5^ cells/mL and treated with 100 ng/mL PMA (Sigma) for 48 hours and then were transfected with siRNA (Santa Cruz Biotechnology) that are specific for FURIN and control siRNA(Santa Cruz Biotechnology) for 24 hours, and this was followed by treatment with 1 μg/mL LPS (Escherichia coli O55:B5, Sigma) and 5 mmol/L ATP (Solarbio) for 30 minutes. The cells were then harvested and used to extract total RNA and protein.

### 2.6 Western blot

For western blotting, cells were washed twice with precooled PBS. Proteins were extracted with RIPA lysis buffer (50 mmol/L Tris and pH 7.4, 150 mmol/L NaCl, 1% Nonidet P‐40, 0.5% sodium deoxycholate; 0.1% sodium dodecyl sulfate (SDS)), collected, and centrifuged at 13 800 × *g* for 15 minutes; the concentration of proteins in the supernatant was determined with a bicinchoninic acid assay (Beyotime biotechnology). Samples of up to 15 μg were electrophoresed on 10% SDS‐PAGE gels and blotted onto a polyvinylidene difluoride (PVDF) membrane. After blocking with QuickBlock™ Blocking Buffer (Beyotime biotechnology), the membranes were incubated with antibodies for overnight at 4°C. Anti‐human FURIN antibody was diluted at 1:500 (sc‐133142, Santa Cruz Biotechnology), anti‐GAPDH (#5174, Cell Signaling Technology), anti‐caspase‐1 (#2225, Cell Signaling Technology), and anti‐IL1β (#12242, Cell Signaling Technology) were diluted to a concentration of 1:1000. The membranes were then washed three times with 0.1% (v/v) Tween‐20 PBS 1×(T‐PBS). The membranes were then incubated with HRP‐conjugated secondary antibody (Huabio, China) for 90 minutes. The proteins recognized by the antibodies were determined by using a chemiluminescence HRP substrate (Millipore Corporation). The concentration of samples in bands was determined according to their fluorescence intensities using a fluorescence scanner and analyzed with the ImageQuant LAS 500 system (Thermo).

### 2.7 Statistical methods

SPSS version 22.0 (SPSS Inc) was used for statistical analyses, and figures were prepared by using GraphPad Prism 5.0 (GraphPad). Consecutive variables were represented with medians (interquartile range, 25th‐75th). The difference between the RA group and healthy group was tested by using Mann‐Whitney *U* test, and the differences among the four RA groups were assessed by using the Kruskal‐Wallis H nonparametric test. Student's *t* test was used for the cell line experiment. Spearman correlation was used to evaluate the linear relationship between FURIN and each inflammatory factor. The statistical power of the study analyses was conducted by using NCSS PASS‐11 software. *P* < .05 was considered statistically significant.

## 3 RESULTS

### 3.1 FURIN expression was significantly higher in RA patients than in healthy control participants

This study included 108 RA patients and 39 healthy control participants. Among the group of RA patients and healthy control participants (69.4% and 69.2%, respectively). The median ages of the RA patients and healthy control participants were 58.0 (48.0‐63.0) and 61.0 (51.0‐67.0) years. There were no significant differences in sex and age between the two groups (*P* > .05). All RA patients were divided into four groups according to the DAS28 scoring. There were 29 remission patients (DAS28 ≤ 2.6), 13 patients with low disease activity (2.6 < DAS28 ≤ 3.2), 38 patients with moderate disease activity (3.2 < DAS28 ≤ 5.1), and 28 patients with high disease activity (DAS28 > 5.1). The percentage of females in each group was 62.1%, 76.9%, 68.4%, and 75.0%, respectively. The median age of each group was 52.0 (45.5‐62.5), 61.0 (44.0‐68.5), 58.0 (49.8‐65.3), and 62.0 (55.3‐63.0) years, respectively (Table 2).

**Table 2:** Demographic and clinical characteristics of RA patients (n = 108)

parameters	RA patients n = 108	Healthy controls n = 39	*P* value^#^	DAS28 ≤ 2.6 n = 29	2.6 < DAS28 ≤ 3.2 n = 13	3.2 < DAS28 ≤ 5.1 n = 38	DAS28 > 5.1 n = 28	*P* value^*^
Female n (%)	75 (69.4)	27 (69.2)	.415	18 (62.1)	10 (76.9)	26 (68.4)	21 (75.0)	.125
Age (y)	58.0 (48.0‐63.0)	61.0 (51.0‐67.0)	.291	52.0 (45.5‐62.5)	61.0 (44.0‐68.5)	58.0 (49.8‐65.3)	62.0 (55.3‐63.0)	.150
Disease duration (y)	5.0 (2.0‐14.0)	—		4.0 (3.0‐8.5)	3.0 (1.8‐6.5)	8.0 (2.8‐16.0)	4.0 (1.8‐11.0)	.255
ESR (mm/h)	25.0 (13.0‐51.0)	—		10.0 (4.5‐13.0)	32.0 (23.5‐38.0）	26.5 (18.3‐51.0)	71.5 (41.5‐87.8)	.000
CRP (mg/L)^a^	4.0 (1.3‐13.1)	—		1.3 (0.6‐2.6)	4.3 (1.8‐8.2)	4.2 (1.4‐11.5)	23.6 (3.2‐59.6)	.000
RF (ku/L)^b^	72.6 (28.5‐220.2)	—		71.7 (41.3‐158.3)	132.9 (46.8‐366.3)	69.7 (42.0‐197.3)	194.9 (61.0‐427.4)	.044
RF positive n (%)^b^	92 (92.0)	—		22 (75.9)	13 (100.0)	33(94.3)	24 (92.3)	.121
Anti‐CCP positive n (%)^c^	70 (72.9)	—		14 (48.3)	9 (69.2)	25 (75.8)	22 (88.0)	0.072
Medicine use n (%)								
DMARDs	90 (83.3)	—		25 (86.2)	12 (92.3)	31 (81.6)	22 (75.9)	.410
Methotrexate	73 (67.6)	—		21 (72.4)	8 (61.5)	26 (68.4)	18 (62.1)	.880
Sulfasalazine	18 (16.7)	—		7 (24.1)	3 (23.1)	6 (15.8)	2 (6.9)	.338
Leflunomide	47 (43.5)	—		10 (34.5)	6 (46.2)	17 (15.7)	14 (13.0)	.685
NSAIDs	33 (30.6)	—		7 (10.3)	3 (23.1)	12 (31.6)	11 (37.9)	.519
Prednisolone	21 (19.4)	—		3 (10.3)	5 (38.5)	7 (18.4)	6 (20.7)	.205
Chinese medicine	23 (21.3)	—		9 (31.0)	0 (0.0)	7 (18.4)	7 (24.1)	.137
No treatment	5 (4.6)	—		0 (0.0)	1 (7.7)	3 (7.9)	1 (3.4)	.480

Note

Data were described as n (%) or median (interquartile range, 25th‐75th). DAS28 28 joint count Disease Activity Score Using ESR.

Abbreviations: anti‐CCP, antibodies to cyclic citrullinated peptides; CRP, C‐reactive protein; DMARDs, disease‐modifying antirheumatic drugs; ESR, erythrocyte sedimentation rate; NSAIDs, non‐steroidal anti‐inflammatory drugs; RF, rheumatoid factor.

^a^ CRP data were found for 93 of RA patients due to a lack of CRP data in fifteen patients.

^b^ RF data were found for 100 RA patients due to a lack of RF data in eight patients.

^c^ 70 anti‐CCP positive was found in 96 RA patients due to a lack of anti‐CCP data in twelve patients.

^#^ Comparison between RA patients and healthy.

* Comparison among the four RA groups.

**FIGURE 1:**
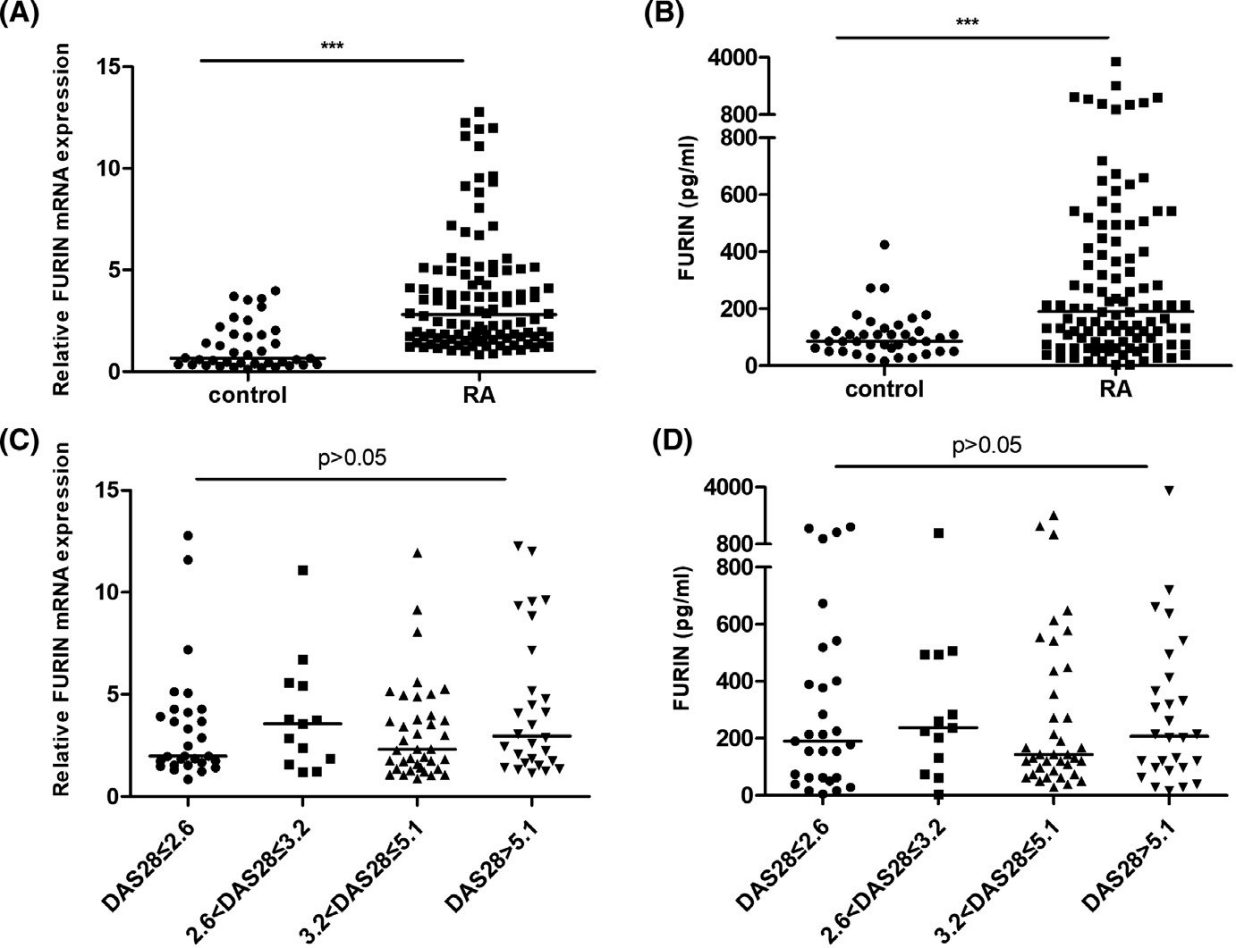
FURIN levels in patients with rheumatoid arthritis and in healthy control participants. A, Relative FURIN mRNA expression in peripheral blood mononuclear cells (PBMCs) from RA and healthy control participants, normalized against the expression of glyceraldehyde‐3‐phosphate dehydrogenase (GAPDH). B, Levels of serum FURIN in patients with RA compared with those of healthy control participants. C, Relative FURIN mRNA expression in PBMCs among four RA groups, normalized against the expression of GAPDH. D, Serum FURIN levels among the four RA groups. Horizontal lines represent the median values in each group. The Mann‐Whitney *U* test was used for statistical comparisons. The differences among the four RA groups were assessed by using the Kruskal‐Wallis H nonparametric test. Significant differences are marked with asterisks: *P < .05, **P < .01, and ***P < .001

FURIN mRNA expression levels in PBMCs were significantly upregulated in RA patients compared to those of healthy control participants (*P* < .001, Figure 1A). Serum FURIN levels were significantly higher in RA patients than in the healthy controls (*P* < .001, median 190.6 pg/mL vs. 85.9 pg/mL, Figure 1B). However, both FURIN mRNA and protein levels were not significantly different among the four RA disease activity groups (*P* > .05, Figure 1C,D). The statistical powers of serum and mRNA levels of FURIN between 108 RA patients and 39 healthy control participants were 0.997 and 1.000, respectively.

### 3.2 Correlation of serum FURIN levels with cytokine levels and clinical laboratory data

**FIGURE 2:**
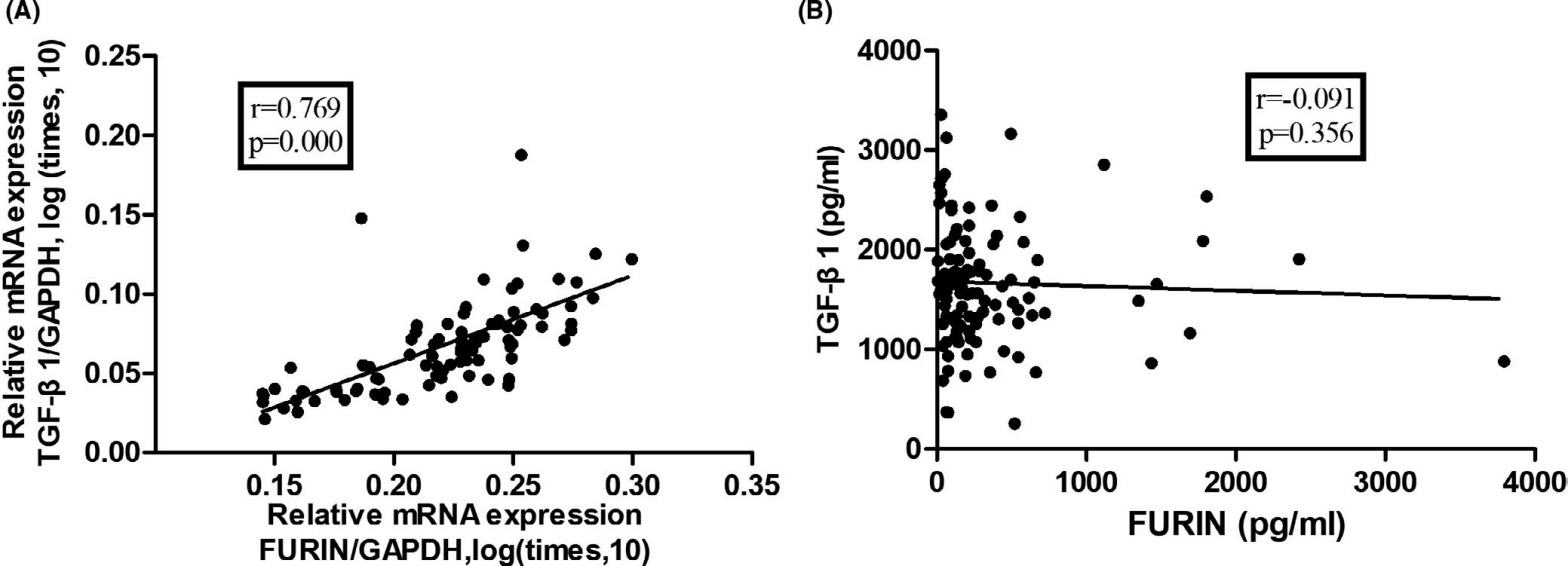
Correlation of FURIN with transforming growth factor (TGF)‐β1. A, Correlation between FURIN mRNA levels and TGF‐β1 mRNA levels, normalized to the expression levels of glyceraldehyde‐3‐phosphate dehydrogenase (GAPDH). B, Correlation of protein levels between FURIN and TGF‐β1 in serum

Studies have reported that FURIN is involved in cytokine secretion and that FURIN is an important enzyme in TGF‐β1 maturation. We analyzed the relationship of FURIN with TGF‐β1 and other cytokines. As shown in Table 3 and Table S1, FURIN mRNA expression was found to be closely correlated with TGF‐β1 in PBMCs (*r* = .769, *P* = .000**)**. But no correlation was observed in serum protein level (*r* = −.091, *P* = .356, Figure 2). FURIN was not correlated with the concentrations of TNF‐α (*r* = −.084, *P* = .404), IL‐4 (*r* = −.096, *P* = .349), and IL‐6 (*r* = .048, *P* = .638). In addition, there was a trend toward a correlation between FURIN and IL‐1β (*r* = −.189, *P* = .059) and IL‐10 (*r* = −.186, *P* = .062). We found that serum FURIN was positively correlated with RF (*r* = .421, *P* = .000) and anti‐CCP (*r* = .294, *P* = .004). FURIN levels were not associated with the serum concentrations of ESR, CRP, C3, C4, or other immunological markers .

**Table 3:** Correlation between FURIN and disease activity and clinical data

Parameters	*r*	*P*
DAS28	.004	.965
Disease duration (y)	.052	.634
ESR, mm/h	.028	.774
CRP, mg/L	.046	.660
RF, KU/L	.421	.000
Anti‐CCP, U/L	.294	.004
IgG, g/L	−.048	.649
IgA, g/L	−.044	.680
IgM, g/L	−.010	.924
C3, g/L	−.007	.947
C4, g/L	−.145	.162
IL‐1β, pg/mL	−.189	.059
TNF‐α, pg/mL	−.084	.404
IL‐4, pg/mL	−.096	.349
IL‐10, pg/mL	−.186	.062
IL‐6, pg/mL	.048	.638

Note

The analysis was conducted by Spearman correlation.

### 3.3 IL‐1β was upregulated in THP‐1‐derived macrophages through the inhibition of FURIN

**FIGURE 3:**
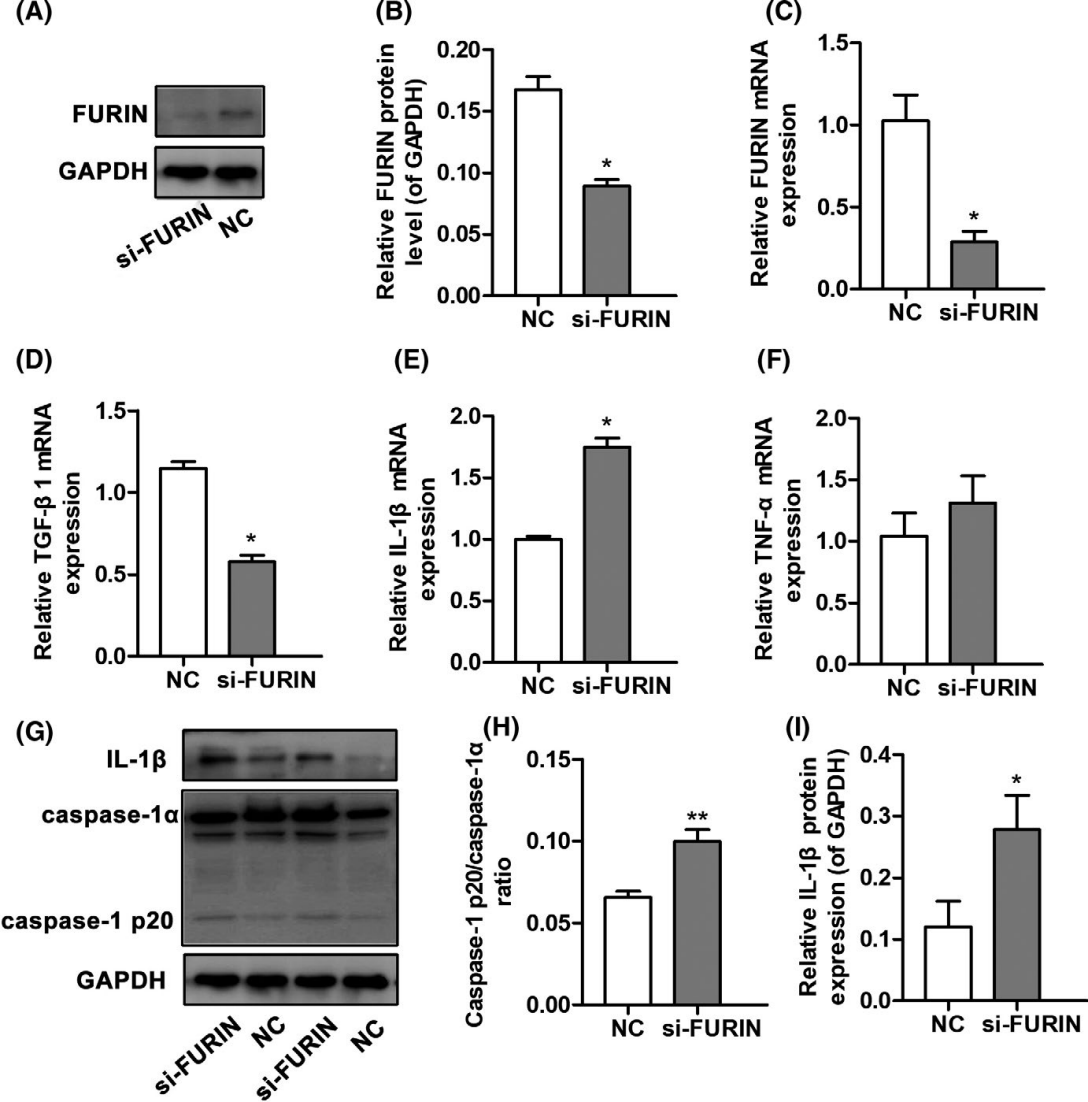
FURIN inhibition in THP‐1‐derived macrophages upregulated IL‐1β and caspase‐1. A, Western blot of THP‐1‐derived macrophages transfected with either FURIN siRNA (si‐FURIN) or control siRNA (NC) for 24 h. B, FURIN protein levels. C, FURIN mRNA levels. D, TGF‐β1 mRNA levels. E, IL‐1β mRNA levels. F, TNF‐α mRNA levels. G, Western blot of THP‐1‐derived macrophages transfected with either FURIN siRNA (si‐FURIN) or control siRNA (NC) for 24 h, followed by incubation with LPS (1 μg/mL) for 4 h and ATP (5 mmol/L) for 30 min. (H) Caspase‐1 protein levels; (I) IL‐1β protein levels. * *P* < .05, **P < .01

To investigate the effects of FURIN on cytokine secretion in THP‐1‐derived macrophages, we used siRNA to interfere with the expression of FURIN (si‐FURIN group) that could then be used to detect changes in the levels of several cytokines, including TNF‐α, IL‐1β, and TGF‐β1. The mRNA and protein levels of FURIN in THP‐1 macrophages were analyzed after siRNA interference 24 hours. As a result, both protein and mRNA levels of FURIN were reduced (*P* < .05). Compared to the normal control (NC) group, TGF‐β1 expression levels were lower in the si‐FURIN group. The expression levels of IL‐1β were increased (*P* < .05). Moreover, we found that the inhibition of FURIN increased the levels of caspase‐1 p20/caspase‐1α (*P* < .05). However, there was no significant difference in TNF‐α levels between the two groups (*P* > .05), as shown in Figure 3.

## 4 DISCUSSION

In this study, we demonstrated that the levels of both FURIN protein and mRNA in PBMCs were significantly higher in RA patients than in healthy control participants. There was no correlation between FURIN and DAS28. In addition, the inhibition of FURIN in THP‐1‐derived macrophages resulted in increased levels of the inflammatory cytokine, IL‐1β.

Consistent with a previous study,^14^ we corroborate that the levels of FURIN are upregulated in RA patients. FURIN is overexpressed in FLS and plays a role in maintaining the homeostasis of rheumatoid FLS.^9^ An association was found among FURIN, RF, and anti‐CCP. Some studies have implicated that FURIN has a role in splenic red pulp, a region enriched with macrophages, that regulates myelopoiesis, cross‐presentation of antigens and tolerance to self‐antigens.^15^, ^16^ Lisi S et al^17^ observed that FURIN is increased in healthy human salivary gland epithelial cells after treatment with human anti‐Ro/SSA antibodies. Thus, we speculated that the autoantibodies, such as RF and anti‐CCP, might also stimulate the expression of FURIN.

In our study, no correlation was observed between FURIN and disease activity in RA patients; this is consistent with a current research suggesting that FURIN levels in peripheral blood were not associated with ESR and CRP. Valli et al^18^ demonstrated that elevated FURIN expression indicated the severity of RA and that the FURIN levels positively correlate with prednisolone treatment. However, we found that the levels of FURIN in the prednisolone treatment group were relatively lower but with no statistic difference (Table S2). Previous studies have shown that FURIN activates precursor Group X secretory phospholipase A2 (GX sPLA2) into mature GX sPLA2 suppresses glucocorticoid production.^19^ However, the mechanism by which glucocorticoids influence FURIN levels has not yet been reported.

A relationship between FURIN and cytokines has been previously reported. In our study, significant positive correlation was found between FURIN mRNA and TGF‐β1mRNA in patients with RA. In THP‐1‐derived macrophages, the interference of FURIN led to decreased levels of TGF‐β1. FURIN is an important enzyme in the maturation of TGF‐β1 and is a positive regulator of its substrates TGF‐β1, which is dependent on Smad signaling.^20^ TGF‐β1 has also been reported to supplement FURIN activity.^5^ Additionally, a positive feedback loop between FURIN and TGF‐β1 has been reported in synoviocytes, which upregulates “A disintegrin and metalloprotease with thrombospondin motif‐4” (ADAMTS‐4); this eventually leads to the destruction of arthritis.^21^, ^22^ However, a recent report showed that TGF‐β1 suppresses human RANKL‐induced human osteoclast development and bone resorption and identified a potential therapeutic target for RA.^23^ We believe that the opposing effects of TGF‐β1 may be due to difference in the experimental cells used in previous studies.^23^

Macrophages are the major source of IL‐1β and contribute to tissue destruction and pain in RA patients. In this study, LPS and ATP treatment contributed to the inflammatory environment in THP‐1‐derived macrophages, and IL‐1β and caspase‐1 levels were higher following the inhibition of FURIN expression. Previous studies have shown that FURIN is multifunctional.^24^ FURIN knockout mice were more sensitive to challenge, and expression levels of IL‐1β were higher than in wild‐type mice.^16^ Likewise, myeloid cells have been reported to express proprotein convertase FURIN which could inhibit the secretion of cytokine of IL‐1β and attenuated inflammation.^16^ Furthermore, exogenous FURIN reduced local IL‐1β production and enhanced the function of regulatory T cells(T_reg_), revealing the protection of FURIN in RA patients.^12^ Although FURIN inhibited the production of certain cytokines, in this study, both FURIN and IL‐1β were upregulated. Elevated FURIN levels in RA patients may be associated with conditions that arise from an abnormal immune and indicate that FURIN may be have the potential to augment the immune response in RA patients.

The limitations of this study are as follows. First, the study included a relatively small group size of the samples; a large number of samples are essential to further confirm our findings. Second, our samples were limited to Chinese patients who were treated, and further research must be conducted to verify these findings in untreated patients with RA. Third, there was no overexpression of FURIN in THP‐1‐derived macrophages that have more strongly supported the anti‐inflammatory effect of FURIN.

In conclusion, we demonstrated that the levels of FURIN in peripheral blood were higher in RA patients but unrelated with disease activity. FURIN may have an anti‐inflammatory effect by inhibiting IL‐1β, it may be a potential target for inflammatory diseases.

## Supporting information

Tab S1

Tab S2
