# Supplementary material for: Increased FURIN expression in rheumatoid arthritis patients and its anti‐inflammatory effect
Source: J Clin Lab Anal. 2020 Aug 25;34(12):e23530. doi: 10.1002/jcla.23530 (PMC7755791; doi:10.1002/jcla.23530)
Supplement: Supplementary file 1 — Tab S1 [file JCLA-34-e23530-s001.docx]

| parameters | RA patients  n=108 | Healthy controls  n=39 | p value ^#^ | DAS28≤2.6  n=29 | 2.6<DAS28≤3.2  n=13 | 3.2<DAS28≤5.1  n=38 | DAS28>5.1  n=28 | p value ^*^ |
| --- | --- | --- | --- | --- | --- | --- | --- | --- |
| IL-6 (pg/ml) | 33.6 (30.1-38.9) | 32.8 (27.5-36.5 | 0.253 | 33.0 (29.0-36.8) | 38.1 (32.2-39.6) | 34.0 (28.6-41.9) | 32.8 (30.3-36.0) | 0.358 |
| IL-1β (pg/ml) | 62.3 (52.5-72.1) | 60.0 (49.7-69.4) | 0.415 | 59.8 (51.5-68.7) | 67.0(51.0-84.1) | 64.4(54.9-72.6) | 62.9(55.0-71.7) | 0.666 |
| IL-4 (pg/ml) | 36.0 (29.9-41.1) | 35.0 (30.9-42.5) | 0.960 | 33.6 (29.7-40.8) | 37.4 (29.8-46.4) | 36.1 (31.9-40.8) | 35.1 (28.9-40.1) | 0.583 |
| TNF-α (pg/ml) | 54.7 (47.6-60.5) | 50.0 (45.7-55.4) | 0.111 | 50.9 (47.2-60.3) | 53.1 (44.2-62.4) | 54.9 (48.6-60.6) | 55.3 (49.2-62.2) | 0.573 |
| IL-10 (pg/ml) | 546.5 (423.8-660.8) | 449.2 (384.0-556.0) | 0.007 | 490.9 (422.3-626.5) | 558.0 (403.6-757.4) | 551.3(422.7-668.7) | 546.5(437.5-670.7) | 0.820 |
| TGF-β1 (ng/ml) | 1.61 (1.26-1.95) | 1.56 (1.16-1.97) | 0.858 | 1.69 (1.37-2.09) | 1.51 (1.06-1.78) | 1.54 (1.11-1.90) | 1.66 (1.31-2.22) | 0.335 |
| FURIN (pg/ml) | 190.6 (77.2-430.6) | 85.9 (51.2-120.8) | 0.000 | 190.6 (62.8-460.1) | 231.4 (109.2-495.5) | 144.0 (80.1-442.4) | 208.1(97.5-401.2) | 0.961 |

**Supplemental Table 1 Serum FURIN and cytokine levels in RA and healthy health groups**

^#^. Comparison between RA patients and healthy. ^*^. Comparison among the four RA groups.
