# Supplementary material for: Increased FURIN expression in rheumatoid arthritis patients and its anti‐inflammatory effect
Source: J Clin Lab Anal. 2020 Aug 25;34(12):e23530. doi: 10.1002/jcla.23530 (PMC7755791; doi:10.1002/jcla.23530)
Supplement: Supplementary file 2 — Tab S2 [file JCLA-34-e23530-s002.docx]

**Supplement Table 2 The correlation between FURIN levels with medicine treatment**

| parameters | Methotrexate | | Sulfasalazine | | Leflunomide | |
| --- | --- | --- | --- | --- | --- | --- |
| Treatment | Yes (n=73) | No (n=35) | Yes (n=18) | No (n=90) | Yes (n=47) | No (n=61) |
| FURIN (pg/ml) | 155.6(68.6-407.1) | 213.9(120.8-507.2) | 260.6(80.1-560.5) | 184.7(74.3-383.6) | 213.9(120.8-448.3) | 155.6(68.6-424.7) |
| p value | 0.271 | | 0.310 | | 0.438 | |

| Prednisolone | | NSAIDs | | Chinese medicine | |
| --- | --- | --- | --- | --- | --- |
| Yes (n=21) | No (n=87) | Yes (n=33) | No (n=75) | Yes (n=23) | No (n=85) |
| 144.0(68.6-348.5) | 213.9(85.9-448.3) | 167.3(109.2-395.3) | 167.3(109.2-395.3) | 155.6（62.8-720.7） | 202.2（91.7-383.6) |
| 0.161 | | 0.989 | | 0.773 | |

**Supplement Table 2 The correlation between FURIN levels with medicine treatment (continued)**

NSAIDs, non-steroidal anti-inflammatory drugs.
